# Carbon Dots: An Emerging Smart Material for Analytical Applications

**DOI:** 10.3390/mi12010084

**Published:** 2021-01-15

**Authors:** Smita Das, Lightson Ngashangva, Pranab Goswami

**Affiliations:** Department of Biosciences and Bioengineering, Indian Institute of Technology Guwahati, Guwahati 781039, Assam, India; smita@iitg.ac.in (S.D.); ng.lightson@iitg.ac.in (L.N.)

**Keywords:** carbon dots, smart materials, photoluminescence, chemiluminescence, electrochemiluminescence, analytical, optical

## Abstract

Carbon dots (CDs) are optically active carbon-based nanomaterials. These nanomaterials can change their light emission properties in response to various external stimuli such as pH, temperature, pressure, and light. The CD’s remarkable stimuli-responsive smart material properties have recently stimulated massive research interest for their exploitation to develop various sensor platforms. Herein, an effort has been made to review the major advances made on CDs, focusing mainly on its smart material attributes and linked applications. Since the CD’s material properties are largely linked to their synthesis approaches, various synthesis methods, including surface passivation and functionalization of CDs and the mechanisms reported so far in their photophysical properties, are also delineated in this review. Finally, the challenges of using CDs and the scope for their further improvement as an optical signal transducer to expand their application horizon for developing analytical platforms have been discussed.

## 1. Introduction

Since the fortuitous discovery of carbon dots (CDs) in 2004, the interest in these luminescent nanomaterials has been increasing exponentially [1,2]. The CDs are zero-dimensional carbon nanoparticles with a size of less than 10 nm. Compared to the different types of carbon nanomaterials, the CDs have gained enormous attention due to its certain advantages such as inexpensive and easy synthesis, facile surface functionalization, low toxicity, biocompatibility, high water-solubility, tunable luminescence property, and stability at room temperature [3]. Consequently, their applications are explored in bioimaging, drug delivery, and chemical/biological sensing [4]. Additionally, carbon dot’s enzyme-like activity has also attracted wide interest because of their potential to replace the naturally occurring enzymes, as the latter is ordinarily unstable, expensive, and challenging to synthesize on a large scale [5].

At present, the concept of employing CDs as smart material is of great interest in material and analytical sciences. A material is termed “smart” when it experiences variation in response to a significant external physical or chemical stimulus that includes light, temperature, pH, solvent, stress, and electric and magnetic field [6]. The synthesis and applications of these smart material or stimulus-responsive materials have been among the principal focus of research in recent times. Conventionally, the smart materials that belong to metal alloys, semiconducting materials, and inorganic compounds are widely used in smart technology such as strain sensors, actuators, and robots [7]. Such materials can change the outlook of modern science and technology and engineering at large. However, the traditional smart materials have limitations ranging from harsh synthesis/preparation conditions to high cost. Therefore, biomaterials and polymers have gradually replaced conventional smart materials in various modern technological fields and applications due to their inherent properties like biocompatibility, lightweight, large surface area, transparent, and simple synthesis [8]. In this context, CDs have increasingly appealing research interest as potential smart materials for their wide applications in bioanalytical sciences.

This review highlights the various routes of carbon dots synthesis, functionalization, surface passivation, and incorporation of heteroatoms to understand the formation of different types of CDs followed by various techniques for characterization. In the subsequent section, we discussed the properties of CDs depending on the route of synthesis, precursor moieties, and heteroatoms addition. The physical and chemical properties of CDs like absorption, photoluminescence, electron transfer, cytotoxicity, and photostability are also extensively focussed. We emphasized the photoluminescence mechanism of CDs, followed by the description of CD’s recent development as smart materials that are responsive to external stimuli such as pH, temperature, light, pressure, solvent, phase, etc. Emphasis has been laid on the application of CDs in different optical-based detection systems covering photoluminescence, chemiluminescence, and electrochemiluminescence.

## 2. Synthesis of CDs

CDs being the rising star of fluorescent carbon nanoparticles have captivated the interest of the researchers for their application in various fields. Depending on the structure, CDs are classified as graphene quantum dots (GQDs), carbon nanodots (CNDs), and polymer dots (PDs). GQDs have graphene layer/s that can be connected with functional groups. CNDs are spherical shaped, and they can be further divided into carbon nanoparticles and carbon quantum dots based on the crystal lattice. PDs are formed due to aggregation or cross-linking of polymers [9]. Therefore, different CDs may be devised and engineered to obtain the desired property by following various synthetic routes.

Diverse methods for the synthesis of CDs are known. Typically, the synthesis process can be grouped into two: Top-down and bottom-up [10]. The top-down approach usually follows breaking-down/cleaving larger carbon molecules to smaller molecular structures, whereas the bottom-up approach involves building-up/step-wise chemical fusion of small carbon molecules by pyrolysis or carbonization. Some known top-down approaches include electrochemical/chemical oxidation, laser ablation, and ultrasonic treatment, whereas the bottom-up approaches include hydrothermal/solvothermal, microwave-assisted, thermal decomposition, carbonization, pyrolysis, and ultrasonic treatment. Some prominent reports on the synthesis of CDs via these two routes are mentioned in Table 1.

The different physicochemical properties like the degree of carbonization, crystallinity, size, morphology, and photoluminescence properties are attained depending on the type of synthesis method and the precursor molecule. For instance, the hydrothermal method is less harsh than the pyrolytic method, but the former generally yields incomplete carbonization, molecular fluorophores, amorphous CDs, whereas the latter predominantly yields graphitic core structures. The functional groups like amine, carboxyl, and hydroxyl on the surface of carbon dot can modify the physicochemical properties, biocompatibility, and stability, thus influencing the selectivity, and sensitivity of the CDs in the analytical applications. Additionally, the molecular precursors used for the synthesis of CD influences the degradation process and other environmental behaviour [31].

As stated before, various ranges of CD materials with different properties could be achieved by different synthesis conditions. It has been recently observed that if bottom-up synthetic approaches are followed, high PL quantum yield (QY) results. Due to the harsh conditions being applied in the bottom-up synthesis approach, multiple side-products may be produced along with the CDs. Citrazinic acid and other small fluorophore derivatives are produced while synthesizing the nitrogen-doped CDs from citric acid, contributing to the emission in the blue spectral range. Such molecular species and polymerized nanoparticles may attach to the CDs to enhance the optical properties [32,33].

### 2.1. Heteroatoms Doped-CD Synthesis

The inherent properties of the CDs can be modulated by doping with heteroatoms such as nitrogen (N), phosphorus (P), sulphur (S), boron (B), and fluorine (F), individually or in various combinations during the synthesis. The introduction of the heteroatoms further adds new electronic, chemical, and optical functionalities to the CD, thereby broadening its application. Depending on the type of element, doping is categorized into non-metal and metal doping.

#### 2.1.1. Non-Metal Doping

Nitrogen-doped (N-doped) CDs are the most commonly explored type of non-metal doping because of the closer electronic structure with that of carbon. The N-doped CDs are fabricated by introducing the nitrogen element to the carbon dot core. For the first time in a study, multicolour emissive CDs were synthesized using inorganic ammonium salt via a solvothermal method. The synthesized CDs with increased nitrogen content resulted in high quantum yield of ~4% and led to the introduction of new functional groups like cyano. However, because of the carbon dot’s multicolour emissive property, these CDs have also opened up various applications for sensing and antibacterial activity [34]. The N-doped CDs developed by using *Lantana camara* berries via a simple green approach exhibited good quantum yield (QY) of 33.15%, high stability, high biocompatibility. Additionally, the N-CDs could also detect Pb^2+^ with high sensitivity and selectivity [35]. Red emissive N-doped CDs was synthesised from citric acid and ethylenediamine through a microwave-assisted pyrolysis method. During the synthesis, the effect of the molar ratio of ethylenediamine to citric acid, pyrolysis time, and the concentration of the reactants was taken into consideration. The optimized molar ratio between amine/acid was found to be 0.75 with an exposure time of 88 s in 1100 W microwave power. The fabricated N-doped CDs displayed remarkable biocompatibility and can further be used in bioimaging applications [36]. In most instances, the CDs synthesis with co-doping enhances the properties of the nanomaterial. Interestingly, human fingernails as precursor was used to fabricate N, S co-doped CDs through a simple microwave irradiation procedure that exhibited good stability and a QY of 42.8%. More importantly, the method does not require any passivating agent or a passivation step for the synthesis. The N, S co-doped CDs were employed to detect a synthetic dye, sunset yellow and in cell imaging [37].

#### 2.1.2. Metal Doping

Doping CDs with metal ions can modify the charge density and surface properties, thus effectively tuning the optical features of the nanomaterial. For instance, the Cu-doped CDs exhibited enhanced peroxidase-like activity due to the enhancement of the electronic properties and modification of the surface properties after the introduction of the metal ion [38]. In another study, the developed Cu-doped CDs using ethanediamine and copper chloride dihydrate (CuCl_2_·2H_2_O) as precursors possessed notable photostability for up to 60 min. Then again, manganese doped CDs (Mn-CDs) displayed excellent fluorescence QY (>54.4%). By using spectroscopic techniques and DFT-based frontier orbital calculation, it was found that the high QY is due to the change of the oxidation state of Mn from Mn-oxide to Mn-carbonate during the synthesis [39]. Some more examples of metal-doped CDs are given in Table 2 for further reference.

### 2.2. Surface Passivation and Functionalization of CDs

Apart from doping, the intrinsic properties of the CD can be effectively tuned during their synthesis by tailoring the surface chemistry via surface passivation/functionalization. During the process of surface passivation, functional groups like -OH, -COOH, and -NH_2_ are introduced onto the surface of the CDs, which further enhance the physical property such as solubility and specific chemical reactivity in general as well [60]. In a study, Bai et al. demonstrated that the polydopamine (PDA) passivated CDs exhibited triple times more QY than the original CDs in the absence of PDA and also displayed 1.5 times enhancement of nucleation site for CD formation [61].

The surface functionalization of the CDs with different precursors such as L-cysteine (N, S), ethylenediamine (N), and glycine (N, O) resulted in high fluorescence QY and improved selectivity for different metal ion detection due to different binding abilities [62]. Understanding the interaction between the CD system and the passivating agents is crucial to tailor the CD’s fluorescent properties. For example, the synthesis routes influence the CD-polymer nanoparticle system’s interaction, that ultimately influencing the photoluminescence property [63]. Ions like Zn^2+^ was also used to develop passivated CDs using zinc gluconate by pyrolysis method of synthesis. The presence of Zn^2+^ during the synthesis prevents the aggregation and improves the stability and optical property of the synthesized CD [64].

The passivation methods and surface functionalization are followed not only to produce better stability and improve the PL intensity but also to enhance other vital characteristics of CDs. Chirality is very important in terms of catalysis, chiral recognition, and even sensing. Recently, Rao and the group developed a strategy using the surface passivation method to prepare chiroptical carbon quantum dots via two steps pyrolytic route. They used tartaric acid and citric acid as carbonaceous sources while producing carbon core, whereas D/L-penicillamine molecules were used to surface passivating agent. In order to retain the chiral information, one of the critical findings of the study was to maintain the reaction temperature lower than the melting point of the ligand in the second pyrolytic step. Interestingly, the strategy did not depend on the chirality of the carbon source [65]. Another interesting study is the effects of surface functionalities of carbon dot during cellular uptakes. Here, the CDs which were passivated with two chemical moieties, 3-ethoxypropylamine (EPA-CDs) and poly(ethyleneimine) (PEI-CDs) were considered to evaluate their uptake mechanism, pathways, and effects in HeLa cells (human cervical carcinoma cells). As expected, the internalization pathways of the two CDs were different in the HeLa cells; however, the efficiency of the internalization process with PEI-CDs was higher in comparison to the EPA-CDs. The different moieties present on the surface of CDs could affect the overall behaviours of CDs uptake [66].

### 2.3. Characterization of CDs

The unique and typical properties of CDs are exhibited due to their size, shape, chemical skeleton/composition, and structure. So, there have been continuous attempts to explore robust and reliable techniques for their characterization. In this section, some of the updated characterization techniques for CDs are discussed briefly as the characterization of CDs has been discussed more in recent reviews and chapters [67,68,69].

#### 2.3.1. Microscopic and Diffraction Technique

Non-destructive imaging and microscopic techniques are used to study morphology and different dimension of nanosized particles. Some of the techniques which are being routinely explored for the measurements of CDs are transmission electron microscopy (TEM), scanning electron microscopy (SEM), atomic force microscopy (AFM), X-ray diffraction (XRD).

The morphology, size distribution, or particle size of carbon dots can be investigated using TEM and SEM. These techniques can be used to determine agglomeration or dispersion of the prepared particles as well. SEM technique is employed to investigate if the particle size ranges from 1–20 nm, and in case if the measurement exceeds the resolution of SEM, TEM, which could offer better-resolving power, is more advised. Nowadays, high-resolution TEM (HRTEM) is extensively used to study the structure and crystalline nature. To understand dimensional surface images of CDs, AFM is being used to obtain 2D images and 3D information of the surface morphology and topography of CDs. Depending on their diffraction patterns, crystalline materials can be characterized by XRD. Thus, the particle size, crystal structure, and purity of carbon dots are investigated through this technique. It is a valuable characterization technique to obtain crystallite features, but the technique could also be used to study amorphous CDs [9,70].

#### 2.3.2. Spectroscopic Technique

Spectroscopic techniques can characterize the synthetic features of carbon dots. Some of the commonly used spectroscopic tools are ultraviolet-visible spectroscopy (UV-Vis), Fourier transform infrared spectroscopy (FTIR), nuclear magnetic resonance spectroscopy (NMR), photoluminescence spectroscopy (PL), and Raman spectroscopy.

FTIR determines and identifies the functional groups such as carbonyl (-C=O), amine/amide (-NH_2_/-CN), hydroxyl (-OH), ether/epoxy (-O-), and others that are present on the surface of the CDs. The presence of moieties and heteroatoms in CDs such as boron (B), nitrogen (N), sulphur (S), silicon (Si), and phosphorus (P) could be identified using such technique. The fine structure information of metal-doped CDs such as aluminium (Al), nickel (Ni), or magnesium (Mg) can be further characterized by X-ray photoelectron spectroscopy [67].

Another important spectroscopic technique is Raman spectroscopy, a non-destructive and non-invasive method to identify the CDs state. Generally, Raman spectra of carbon dots have two first-order bands, i.e., D- and G-band. D bands signify the vibration of carbon atoms of disordered graphite or glassy carbon, whereas the G band represents the vibration of sp^2^ carbon atoms. The degree of purity or graphitization of CDs could be estimated by the ratio of D band and G band (D/G). A high D/G ratio indicates the amorphous nature, and a high degree of graphitization gives a relatively lower D/G ratio.

NMR usually employs to understand the structural information of CDs further. The presence of sp^2^ carbon, sp^3^ carbon, functional groups like -C=O, -NH_2_, and -OH can be distinguished with the resonance spectroscopy. ^13^C-NMR could be used to distinguish further and confirm the presence or absence of aliphatic carbon or aromatic carbon on the CDs skeleton. The optical properties of CDs are extensively investigated with the help of UV-Vis and PL spectroscopic techniques. The absorption and the photoluminescence properties of CDs are explained more in the later part of the review.

One of the powerful tools to elucidate the chemical structure of smaller-sized nanoparticles is mass spectrometry (MS). Some of the MS techniques which have been applied and used to characterize CDs are electrospray ionization quadrupole time-of-flight tandem mass spectrometry (ESI-Q-TOF-MS/MS), inductively coupled plasma-mass spectrometry (ICP-MS), and matrix-assisted laser desorption/ionization time-of-flight mass spectrometry (MALDI-TOF-MS) [71,72].

## 3. Properties of CDs

CDs are fascinating nanomaterials with remarkable inherent physical and chemical properties that allow their application in diverse fields. Therefore, an in-depth study and understanding of the CD’s optical, physical, and chemical properties are essential to further improve its characteristics in order to broaden its applications.

### 3.1. Physical Property

#### Chemical Structure

Carbon dots usually are less than 10 nm with quasi-spherical nanosized carbon particles. However, as mentioned earlier, synthesis routes dictate the various chemical structure of carbon dots. For instance, GQDs are anisotropic with a crystalline structure of one or more graphene layers. Different analytical techniques such as microscopic, spectroscopic, spectrometric, and diffraction methods are employed to confirm and ascertain the morphology, functional groups, size distribution, and crystalline nature of CDs. For example, the morphology of microstructure CDs-based lubricants following the ultrasonic approach was studied with TEM and HRTEM and found to be 2.38 nm on average and highly crystalline with 0.21 nm lattice spacing and (100) graphene plane [12]. The structural elucidation of defects and or graphitization could be further confirmed with Raman spectrometry by analysing the G band and D band. By following the pulsed laser ablation synthesis method, nitrogen-doped GQDs were prepared and obtained a 3 nm particle size by Neon and co-workers. The as-prepared N-GQDs exhibited 1565 cm^−1^ as G band and 1311 cm^−1^ as D band in Raman spectrum, confirming the disordered structure [40]. XRD is a powerful instrument to characterize the physical state of the synthesized carbon dots. The amorphous character of CDs is observed as a broad hump, which is centred in and around ~2θ = 26° in the XRD profile. Nearly spherical amorphous clusters of ~4–18 nm diameters were observed in the self-passivated CDs from dextrose by following the ultrasonication approach [73].

### 3.2. Chemical and Optical Property

#### 3.2.1. Ultraviolet-Visible Absorption

The basic chemical structure of CDs can be significantly elucidated by the typical UV-vis spectral analysis. The presence of π-π* (C=C) and n-π* (C=O, C-N, C-S, etc.) transition of the CDs skeleton indicates the type of surface functional groups, routes of CD synthesis, precursors, and chemical environment. For example, the absorption bands of CDs at around 273 nm and 342 nm may imply sp^2^ hybridization of the π electrons and n-π* transition, respectively [15]. Again, the combination of the same carbon but different nitrogen sources also influences the absorption’ bands position [56,74]. The presence of heteroatoms (such as N, O, P, B, S, and Se) in the CD’s molecular structure also results in the fluctuations of the UV-vis peaks. The N, S-CDs fabricated from 3-aminothiophenol via a one-pot hydrothermal method showed two absorption shoulders at 298 nm, and 354 nm attributed to n-π* transition and heteroatoms N and S surface states defect, respectively [75].

#### 3.2.2. Photoluminescence

**Figure 1:**
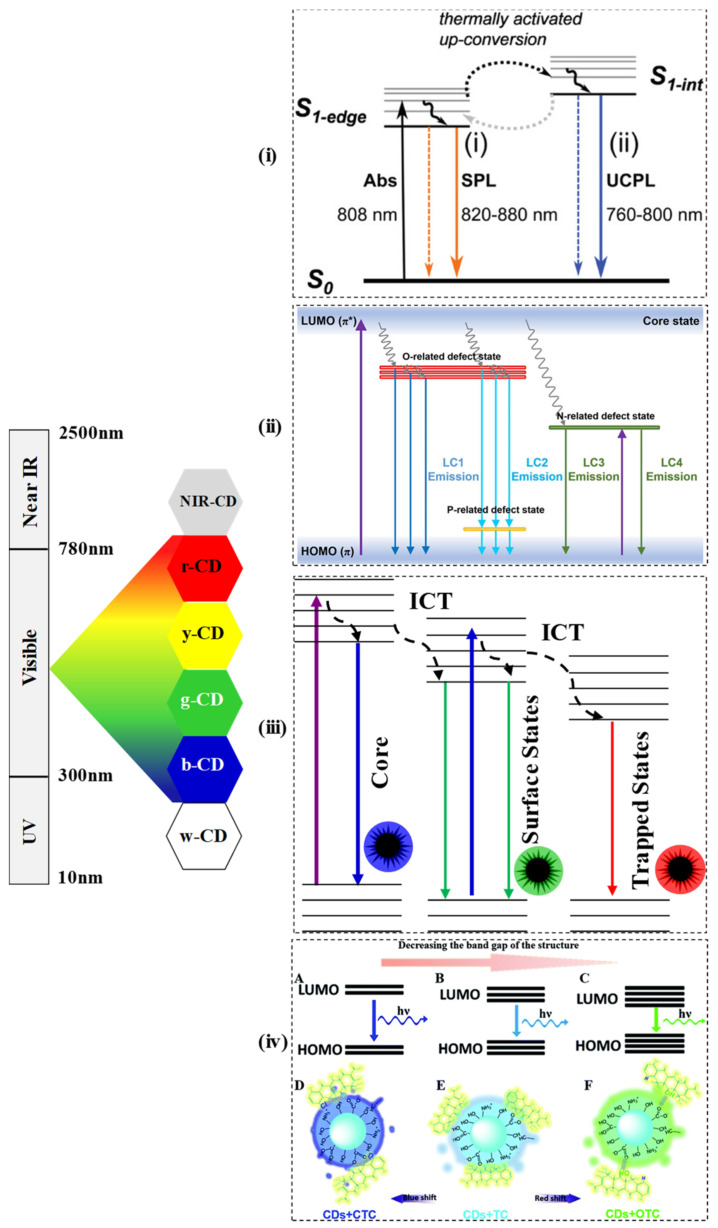
The band gap and transition of various colour exhibited by CDs (near infrared CD: NIR-CD, red-CD: r-CD, yellow CD: y-CD, green CD: g-CD, blue CD: b-CD, white CD: w-CD). (**i**) Proposed mechanism of up-conversion PL in Near IR (NIR) CDs [76] (Reproduced with permission), (**ii**) Proposed mechanism of CDs with O-defects, P-defects and N-defects states [24] (Reproduced with permission). (**iii**) Energy diagram of the CDs indicating the role of intersystem, charge transfer [41] (Reproduced with permission). (**iv**) Band gap transitions of CDs by introducing CTC, TC, and OTC [17] (Reproduced with permission).

The most impressive characteristic feature of CDs is the photoluminescent (PL) or the fluorescent property, which has allowed them to expand their field of applications. The PL of CDs is influenced by surface chemistry, quantum size effect, and molecular states of the carbon core. The upconversion photoluminescence (UCPL) in the near infrared region carbon dots (NIR-CDs) may be because of the thermally activated electron transition from S_1-edge_ to S_1-Int_ in the excited state as shown in Figure 1i [76]. The various synthetic approaches (as shown in Table 1 and Table 2) along with different starting materials result in the generation of CDs with unique structures and different luminescent behaviours such as white, blue, green, yellow, red, and deep ultraviolet emission. Since the CDs are fabricated from variable carbon sources via different routes, the PL behaviour also depends on the size, solvent, pH, and many more. Generally, due to the diverse electronic transition pathways, the CDs exhibit broad, symmetrical luminescence spectra across the whole wavelength scale. Various energy levels may be created by O-containing groups (named as O-related defect state), P-containing groups (named as P-related defect state), and N-containing groups (named as N-related defect state) on sp^2^ hybridized carbons of the synthesized CDs as shown in Figure 1ii [24]. Moreover, compared to the quantum dots and other organic dyes, the CDs usually exhibit large Stokes shifts. The QY of the CDs depends on the surface chemistry and preparation methods. Yellow-emissive CDs from anhydrous citric acid and 2,3-phenanzinediamine were prepared that exhibited large Stokes’s shift (188 nm), excellent stability, and 24% quantum yield [55]. In another study, multicolour PL emissive CDs were synthesized from m-phenylenediamine (m-PD) and o-phenylenediamine (o-PD) and in the presence and absence of tartaric acid as the starting materials. Tartaric acid played a crucial role in tuning the surface state of CDs, such as the increase in surface oxidation and carboxylation. In the presence of tartaric acid (TA), the CDs from m-PD and o-PD exhibited green colour and red colour respectively whereas, in the absence of TA, CDs from m-PD and o-PD exhibited blue colour and yellow-green colour respectively. In particular, the red-CDs exhibited a high QY of up to 22.0%. [77]. The doping of CD changes the excitation-dependent PL. In a study, the emission of a bare CD that was found to be excitation-independent, showed a large red shift when it was doped with nitrogen. The reason is ascribed to the superimposition of blue (intrinsic molecular centres) and green emission bands (extrinsic molecular centres). In the emission process, the energy is transferred from the electron-hole pair formation at the intrinsic centres of the core to the extrinsic surface centres. Due to the surface defects and hybrid nanostructure in the mesoporous matrix, the contribution of the two bands modified, enhancing the tunability of the emission, thus promoting the green PL from blue emission. Besides, the QY of the CDs was found to vary, for the bare CDs (1.4%), N-doped CDs (22%), and purified N-CDs (28%) [78]. In another study by Kipnusu and co-researchers, nitrogen and boron doped carbon dots exhibited enhanced intersystem charge transfer (ICT) due to the presence of donor–acceptor moieties. Due to the ICT, the synthesized CDs produced triple colour emission as shown in the Figure 1iii [41]. The properties of PL are controlled by the surface functionalization of CDs as well. Recently, surface functionalization was shown to enlarge and narrow the band gap energy. The fluorescence of synthesized CDs could be quenched by tetracycline (TC) due to inner filter effect, whereas by introducing chlorotetracycline (CTC), blue-shift fluorescence was induced which may be due to enlarged energy band gap, and upon introducing oxytetracycline (OTC), fluorescence experienced red shift which could be because of the narrowed band gap as shown in Figure 1iv [17]. In a recent report, the addition of a long-alkyl chain to the CDs promoted the emission of white luminescence under UV light (365 nm), which may be due to the inhibition of the aggregation-caused quenching effect. Additionally, the alkyl chains can effectively interact with the lipophilic fatty residues that can increase the potential applications of the developed white-CDs [79]. Metal ion-dependent PL quenching of CDs has become a known phenomenon. In a report, the quenching mechanism has been identified as the photoinduced transfer of electrons from amine functional groups of the CDs to the respective metal ions [80].

#### 3.2.3. Electron Transfer of CDs

Typically, CDs can act as both an electron acceptor and donor. In an interesting study, considering the exceptional electron mobility property of the CDs at room temperature, CDs functionalized with ionic liquid were fabricated for their application as nanofluid in the field of energy. The nanofluids comprising of organic/inorganic hybrid systems could be used as electrolytes and separators for energy storage. Highly conductive Cdots, Cdots/[Bmim]Cl/[Tmi][Trif] and CdotsCHI/[Bmim]Cl/[Tmi][Trif], could be entrapped in poly(vinyl alcohol) membrane, which exhibited high proton conductivity. Moreover, the nanofluid’s features remain constant for four months, even on wetting/drying cycles [81]. One of the most common applications of CDs is in the field of photocatalysis. The photocatalytic performance of the graphitic and amorphous CDs, which were synthesized using fructose, glucose, and citric acid, was investigated. In this study, the type of carbon source and the synthesis route that ultimately determines the potential of the photoelectron transfer determined the CD’s structure and optical properties [82]. Pure carbonaceous CDs (without dopants) in graphitic form exhibited better photoactivity than the amorphous one, whereas, in the case of nitrogen-doped CDs, the amorphous CDs exhibited better photoredox-activity due to the presence of photo-active molecule. The graphitic defects and the dopant can quench each other, which reduces the photoactivity [83].

Heteroatom doping in carbon materials has long been used to create active catalytic sites and increases oxygen reduction reactions (ORR) in electrocatalysis. The performance of carbon-based catalysts, composites of doped CD and reduced graphene oxide (CD/rGO), and directly doped rGO were studied. In the finding, CD/rGO outperformed in ORR measurement to their corresponding counterparts, and it is noted that N, S-co-doped too performed better than the individual doped N-CDs or S-CDs. Some of the reasons for such behaviour could be the synergistic effects of N, S-co-doping providing four-electron transfer pathways in ORR, active sites of on the CD surfaces and located at the edges/defects in abundant, which may have more accessibility to oxygen molecules. Another reason could be the effective components like graphitic N atoms and C-S-C/S-N species where current density and half-wave potential could be improved [84].

#### 3.2.4. Cytotoxicity and Photostability of CDs

CDs are mostly known for their fascinating biocompatibility and relatively less toxicity, thus fulfilling the required conditions for diverse applications. The cytotoxicity of CDs, both in vitro and in vivo conditions, are extensively investigated. Moreover, CD’s photostability is a crucial characteristic that can be explored for its efficient use as stable fluorescent probes. As the name suggests, photostability here means the labelled cell’s fluorescence intensity remains stable for a considerable time. With this property, CDs are growingly used in cell imaging. Semi-conductor quantum dots were used in bioimaging methods because of their better photophysical properties than organic chromophores. The use of the conventional metal based QDs for biomedical applications are limited because of toxicity of the heavy metals like Pb and Cd, present in these nanomaterials. Since CDs are free from toxic heavy metals and possess high photostability, these are used in the bioimaging fields and medical diagnosis as well. However, a report of low toxicity of metal chelated CQD is available. A CQDs from citric acid and polyethylenimine was synthesised and covalently conjugated with 1,4,7,10-tetraazacyclononane (DOTA) to chelate lanthanide ion (Ln = Eu, Tb, Yb, and Gd). The CQDs-DOTA-Ln exhibited low cytotoxicity against Hela cells even at its high concentrations (500 µg/mL) [85]. However, the synthesis of metal-doped CDs is time-consuming, complicated, involves multiple steps, and requires post-synthetic treatments. In a recent development, ruthenium-containing CDs (Ru-CDs) were fabricated using a simple and efficient strategy. The developed CDs exhibited enhanced red fluorescence compared to the bare CD and Ru-complex and were employed as bioimaging agents for tumour cells and as photodynamic nanoagents for cancer therapy [86].

#### 3.2.5. Emerging Property: Chirality of CDs

More recently, efforts have also been made to develop chiral carbon dots for their application in a myriad of exciting areas ranging from sensing of chirality, separation of chiral molecules, chiral catalysis, bioimaging, and biomedicine. Among the two types of CD synthesis approaches, the “bottom-up” approach usually generates better chiral CDs as the precursor molecules themselves are chiral and therefore do not need the introduction of chiral ligands during the synthesis process [87]. A hybrid CD/CNC (cellulose nanocrystal) nanoparticle synthesized via hydrothermal route showed a dissymmetry factor of 0.2, which is higher than that of the reported dye/CNC hybrids. Furthermore, the nanoparticle displayed higher left-handed circularly polarized luminescence (CPL) emission than right-handed CPL emission. Based on the significant findings, these hybrids have a promising application to remove autofluorescence drawbacks in bioimaging. The CD/CNC hybrids can also be effectively employed in sensing, drug delivery, and photonic applications as mirror-free cholesteric lasers [88]. In another interesting study, using the concept of donor–acceptor complex formation between CDs and porphyrins, the chirality of the carbon nanodots was transferred to the porphyrin. This experimental finding provided the possibility of forming chiral composites for different applications [89]. In another approach to enhance the material property for biomedical applications, chiral CDs derived from glutamic acid were doped into gels as the latter display superior biocompatibility. An important aspect of such a doping process is the requirement of chiral match ability between the CD and the gelator, which would otherwise lead to the disintegration of the gel. It was shown that the doping of the chiral CD with the gelator (N,N-bis(octadecyl)-D-aminoglutamic diamide) to form gel resulted in the fluorescence enhancement of the CD. The enhancement was assumed to be the restriction of Brownian motion in the gel system that otherwise is responsible for decreased fluorescence intensity in the liquid system due to collisions between the CDs [90]. Fluorescence off-on sensors have an enormous demand for the development of smart monitoring systems. Using chiral CD synthesized from citric acid and L-aspartic acid, an on–off and off-on fluorescence sensor was developed to detect both Sn^2+^ ion and L-Lysine enantiomer. While the binding of Sn^2+^ on the surface of the CD resulted in fluorescence quenching (on-off), the addition of L-Lysine to the CD-Sn complex resulted in the enhancement of the fluorescence (off-on) as the L-Lysine preferentially has a stronger binding affinity for Sn^2+^ thereby recovery of the CD’s fluorescence [91].

## 4. PL Mechanism of CDs

Even though the research of CDs is thriving, the exact PL mechanism of the CDs is still in debate. Some of the most acceptable PL mechanisms include quantum confinement and surface states-based explanations. In brief, when semiconductors exhibit properties like size-dependent energy and bandgap transition in the nanometre scale due to the distribution of electrons in the crystal boundary, such behaviour of the materials is known as the quantum confinement effect (QCE). The CDs have a band gap that is non-zero, hence exhibit fluorescence phenomenon under excitation. Additionally, the PL mechanism of CDs could be closely related to surface chemistry, like the presence of functional groups or the surface oxidation states. The diverse surface functional groups such as -C=O, -COOH, -CN, -NH_2_, and -OH can give rise to distinct fluorophores and energy levels. Besides, the surface defects are directly proportional to the extent of surface oxidation that influences the emission wavelength of the CD.

**Figure 2:**
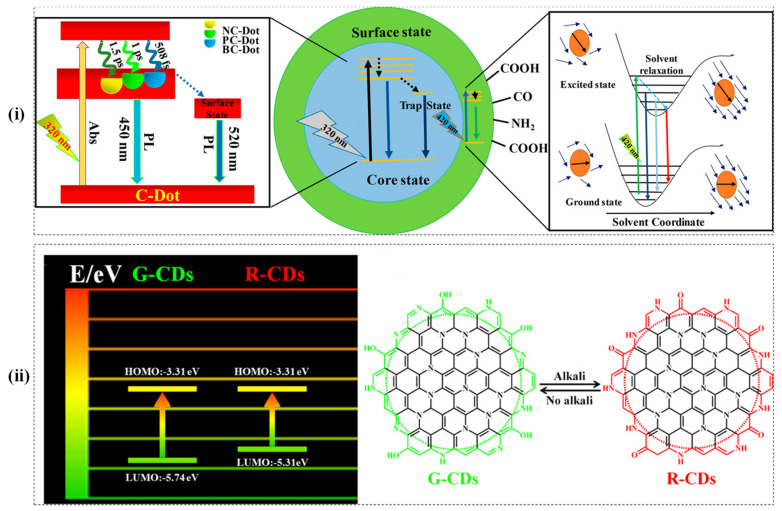
(**i**) Energy levels (HOMO and LUMO) of G-CDs and R-CDs (left side); Luminescence mechanism (right side) [92] (Reproduced with permission). (**ii**) Excited-State for Doped C-Dots at Core State and Surface State, [51] (Reproduced with permission).

In a new development, the reported CDs with high QYs (up to 80%) exhibited green and red reversible switching photoluminescence. The green CDs were synthesized from 3, 4, 9, 10-nitroperylene in an alkaline solution, whereas the red CDs were developed by modifying the surface electronic state of the green CDs by adding alkali. The red emission was due to the narrow band gap, which is further influenced by the surface electronic state, as shown in Figure 2i [92]. In another interesting study, the PL of CD and ionic liquid were found to be similar. The energetically different associated structures in the ground state of the CD govern the fluorescence response [93].

The surface functional groups on CD plays a crucial role in modifying the PL properties. In a report, the CDs from four types of lysine derivatives with different quantities of amino and carboxyl groups were prepared. The CDs with a higher number of functional groups from the precursors resulted in a higher degree of cross-linking, which could lead to fluorescence enhancement and better stability [94]. Besides, the electronic acceptor levels of CDs were influenced by the electronic states of core and ground. The interactions such as hydrogen bonding and affinity behaviour of the CDs could be determined by the chemical nature of the surface groups [95]. In another study, the catalytic activity of the CDs was studied with different surface modifications. Two different CDs were synthesized from poly(ethyleneimine) (PEI-CDs) and citric acid (CA-CDs) which were then used to monitor the peroxidase-like activity in the presence of the substrates like 3,3′,5,5′-tetramethylbenzine (TMB) and 2,2′-azino-bis(3 ethylbenzothiazoline-6-sulphonic acid) (ABTS). Interestingly, the TMB exhibited a higher affinity to the negatively charges CA-CDs, whereas the ABTS showed a higher affinity to the positively charged PEI-CDs [96]. The surface modification controls the optoelectronic behaviour of the CDs like band gap energy, quantum tunnelling, or yield of triplet excitons. Behera et al., studied three different surface states of CDs (oxidized CDs, reduced CDs, untreated CDs) that had not much alteration in the physical dimensions. They noticed that the spectral signature of singlet and triplet excited states and the charge transport properties differed from each surface state. Moreover, this study also emphasized the redox-based surface modification as oxidized CDs exhibited small barrier height compared to the reduced CDs [97]. The influence of CDs bearing different surface amine-derivative ligands on device performance, such as hole transport materials in inverted hybrid LEDs, was studied recently by Paulo-Mirasol’s group. They highlighted that the different surface ligands would affect the optical properties and other parameters like device turn-on voltage and luminance. Besides, varying results were also observed for CDs capped with aromatic ring and nonaromatic ligands [98].

Doping of CDs with non-metallic ions influences the nanomaterial properties, which further affects the PL mechanism. In a study, the dynamics of the excited-state with nitrogen-doped, boron-doped, and phosphorus-doped CDs were investigated. It was observed that the relaxation time profoundly depends on the doping type within the core structure. Further, doping also influences the carbon atom’s charge delocalization that ultimately affects the solvation time as shown in Figure 2ii. Besides, the N-doped CDs exhibited the highest charge carrying capacity, followed by P-doped and B-doped. The breaking of π-π stacking and formation of defects in the P-doped and B-doped CDs was responsible for low charge carrying capacity [51].

Another fascinating new insight of PL is the possible contribution from the small molecular fluorophores. In the recent development, it has been observed that the PL of the CDs could be increased due to the presence of small fluorophore molecules if the bottom-up synthetic route is followed [33,99]. The presence of molecular fluorophores and their aggregates may or may not be CDs and may misguide to understand the structure-property correlation. Such fluorophores could be embedded in between the polymeric carbon dots and thus exhibit enhanced optical properties in absorption and emission spectra due to the possible synergistic effects [99,100]. The impact of such molecular fluorophores may be depending on the arrangement and position with CDs. Langer and co-workers reported 5-oxo-1,2,3,5-tetrahydroimidazo-[1,2-α]-pyridine-7-carboxylic acid (IPCA), a molecular fluorophore formed in CDs derived from citric acid and ethylenediamine, showed a tendency to interact and self-assemble into a stacked like system with the CDs. IPCA could also be incorporated and interacted with the core structure of CDs [101]. Some of the moieties or molecular fluorophores not only enhance the optical properties but also responsible for emission other than the actual CDs emission such as 4-hydroxy-1H-pyrrole[3,4-c]pyridine-1,3,6(2H,5H)-trione (HPPT) [102], and hybrid luminescence in the solution [103].

## 5. CDs as Smart Materials

The rapid progress in materials science over the past few decades has led to the development of smart materials with attractive physicochemical properties bearing great application potential to design next-generation sensing platforms [104]. The smart materials usually include different materials such as metals, nanomaterials, polymers, biomolecules, and even hybrid materials [105,106]. Recently, the carbonaceous materials received increasing attention because of their biocompatibility and non-toxic behaviours. Various carbon-based materials like graphene/graphite and their different derivatives [107,108,109,110], carbon nitride (CN) films [111], carbon fiber tube [112], paper-based bionanocomposites [113] have been studied for diverse applications. Carbon dot or carbon quantum dot is a new addition to this domain of smart materials. These materials are reactive to numerous external stimuli, as discussed below.

### 5.1. pH Sensitive

The detection of pH level is crucial in pathological and physiological processes as well as in the natural environment. Recently, CDs have been explored for their applications as a pH probe due to its intense fluorescence property, excellent photostability, and convertible emission colours. Using a one-pot hydrothermal method, AC-CDs were synthesized from 5-amino-1,10-phenanthroline (Aphen) and citric acid (CA). The resulting CDs exhibited an interesting pH-dependent fluorescent tricolour emission. It is found that Aphen is very sensitive to pH, which may be attributed to the response of the pyridyl group in the presence of H^+^ and OH^−^ medium. The pH-responsive AC-CDs displayed bright green colour at pH 1, grayish at pH 1 to 8 that turned blue to purple from pH 8–13, and finally to yellow at pH 14 [47]. Heteroatom doped CDs (N, S-CDs) synthesized from o-phenylenediamine, L-cysteine, and ethanol also showed excellent pH-response and reversible fluorescence upon pH change. While the fluorescent intensity of N, S-CDs decreased from pH 1.0–13.0 in the basic condition (NaOH), the fluorescence intensity could be reverted from pH 13.0–1.0 using acidic medium (HCl) [114]. Yang et al. developed different sizes of CDs following a simple green methodology of microwave-assisted pyrolysis using xylose, m-phenylenediamine, and H_3_PO_4_ as precursors. In this work, green-emitting CDs were selected, followed by adjusting the CD solution’s pH from 3–11 using PBS buffer. No change was observed in the CD’s excitation/emission at 442 nm/518 nm upon increasing the pH from 3 to 7; however, at pH 8, the excitation/emission shifted to 375 nm/500 nm suggesting the pH-dependent PL property of the CD as indicated by green to blue shift performance. The author proposed that the shifting was dominated by the O-related defect state and P-related defect state, respectively [24]. Another interesting study found that when the anticancer drug doxorubicin hydrochloride (Dox) was loaded with the CDs synthesized from citric acid and urea following the microwave-assisted method, the CDs generated white light from pH 12 to 2 with reversible photo-switching. The role of FRET was likely to be responsible in the white light generation [115]. The pH also affects the emission intensity of the CD. The blue emission of the CD was enhanced as the pH of the solution was moved from 7 to 14. In contrast, the CD’s fluorescence gradually reduced and shifted to longer wavelengths from pH 7 to 1. Thus, the as-prepared N-CDs from citric acid and o-phenylenediamine showed promising pH sensing ability [116].

### 5.2. Temperature Sensitive

**Figure 3:**
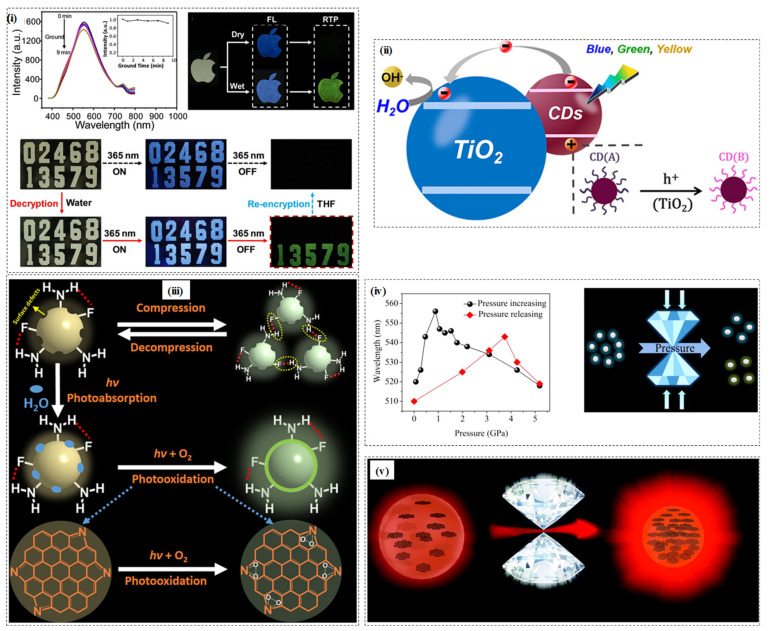
(**i**) Phosphorescent spectra of TA-CDs powder against time, illustration of the CDs with water-stimuli-responsive production and the encryption and decryption process [118] (Reproduced with permission). (**ii**) The photoenergy transformation in the IA-CDs/TiO_2_ nanocomposite system [119]. (Reproduced with permission). (**iii**) Proposed mechanism of the pressure triggered fluorescence [19] (Reproduced with permission). (**iv**) PL peaks when pressure is released or increased (left) and proposed mechanism of piezochromic behaviour of N-CDs (right) [54] (Reproduced with permission). (**v**) Illustration of PL enhancement under high pressure [120] (Reproduced with permission).

There are some reports of temperature-responsive CDs in recent times [117]. With increasing temperature, the fluorescence intensity of the CDs decreased, and vice-versa. The temperature-sensitive property of the synthesized CD was attributed to the dominance of the non-radiative and radiative transitions of the MnOx-CDs at higher and lower temperatures resulting in decreased or increased emission of photons respectively [49]. This feature of the CDs was employed as a thermometer in the HepG2 cancer cell line. The phenomenon was ascribed to thermally activated electronic transitions. Powdered CDs embedded in the trisodium citrate matrix exhibited tunable colour emission from green to yellow. The powdered CD was applied to fabricate white light-emitting devices on blue chips with tunable colour temperature [53]. In another development, the CDs possessing dual-emissive aggregation-induced room-temperature phosphorescence (RTP) behaviour were designed. The CDs were synthesized using trimellitic acid (TA) via a one-pot hydrothermal treatment that emitted white light and yellow light RTP in the solid-state under an on and off UV excitation at 365 nm. While the white light emission was associated with the dual-emissive nature (blue and yellow fluorescence) of the CD, the yellow RTP emission was due to the excited triplet state upon the aggregation of the CD as shown in Figure 3i. The primary goal of the research was to apply the CD in advanced anti-counterfeiting and encryption methodologies [118].

### 5.3. Light Sensitive

Light as a stimulus for CDs to initiate or change various photometric response is a well-known phenomenon. In a recent report, a bidirectional photochromism via anchoring of CDs to TiO_2_ porous films was invented. Under blue light irradiation, the colour of the CDs/TiO2 film obtained by dropping anchoring becomes darker and that obtained by immersion anchoring becomes lighter. The spectral response was intensely dependent on wavelength and polarization of the exciting light for the photobleaching material system. The findings provide a new dimension for optical information encryption and memory. The mechanism of such behaviour has been attributed to the generation of electron-hole pairs from CDs under visible light excitation, followed by the hole’s reaction, which triggered the oxidation reaction with the surface functional group on the CD causing photobleaching as shown in Figure 3ii [119]. In another report, the CDs/ TiO_2_ nanocomposite showed photocatalytic colour switching ability. Upon UV-irradiation, the blue colouration of the methylene blue dyes with the CDs/TiO_2_ nanocomposite could be photobleached within one minute to colourless state. With visible light irradiation, the original blue colour was fully recovered within 20 min [121].

### 5.4. Pressure Sensitive

CDs exhibit piezochromic property wherein an application of 0–22.84 GPa pressure resulted in the change of luminescence colour from yellow to the blue. The pressure-induced change in optical property has been ascribed to the hybridization transition of sp^2^ to sp^3^ in CD. Additionally, such a phenomenon has also paved the way for a better understanding of the optical properties [122]. Taking advantage of the piezochromic effect, a novel N-doped CDs was synthesised that displayed partial reversible piezochromic luminescence. Upon an application of 0.007–5.18 GPa pressure, the red- and blue-shifted photoluminescence (PL) of the synthesized N-CDs could be observed with blue to green colour of PL, whereas when the pressure was released from 5.18 GPa to 1 atm, the red- and the blue-shifted photoluminescence could be tested as shown in Figure 3iv [54]. Geng et al. recognized that in the carbon polymer dots with increasing pressure, the red-shift was because of the increased pi-pi stacking of the pi-conjugated system and the blue-shift resulted due to the hydrogen bonding [123]. In a recent study, a F, N-doped CDs demonstrated a remarkable photoactivated fluorescence enhancement in the presence of continuous UV light and both ambient (1.0 atm) and high pressure (0.1 GPa) as shown in Figure 3iii. Besides, the CDs also showed reversible piezochromic behaviour from 1.0 atm to 9.98 GPa pressure with aggregation-induced enhancement (AIE) in the 1.0 atm-0.65 GPa. The AIE behaviour was because of the increased formation of van der Waals forces and hydrogen bonding among the surface -NH_2_ and -C-F groups on F, N-doped CDs [19]. In this direction, the influence of the solvent on the piezochromic behaviour of CD was also identified as with increasing pressure, the CDs displayed red-and-blue-shift piezochromism with DMF and water as the pressure transmitting medium. The reason being attributed to the π-π stacking, and protic-solvent-induced surface chemical structure changes for the red-and blue-shift effect, respectively [124]. The stability and enhancement of CD’s red fluorescence could be achieved under the influence of external pressure, which may be because of the pressure-triggered aggregation-induced emission. The intensity of the fluorescence decreased when a pressure of above 1.2 GPa was applied, and this phenomenon could be due to the destruction of the molecular structure of R-CDs, as shown in Figure 3v [120].

### 5.5. Multi-Sensitive

**Figure 4:**
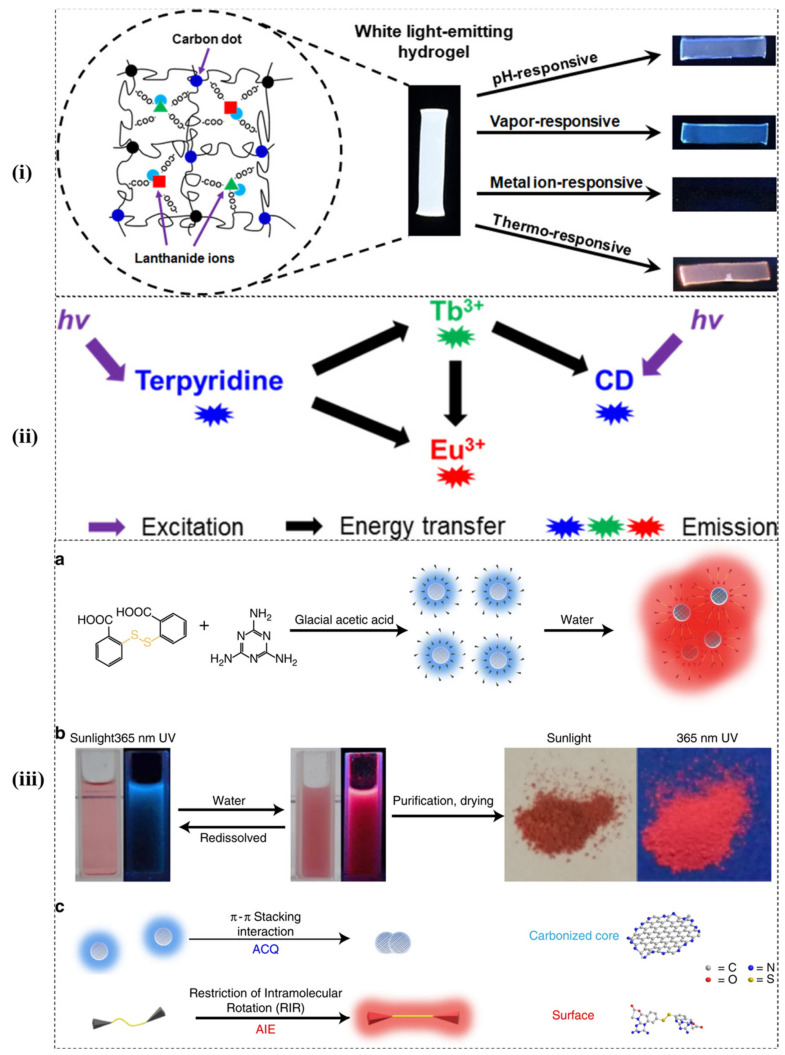
(**i**) Multi-stimuli responsive behaviour of the CDs, (**ii**) Proposed energy-transfer mechanism in white-light-emitting hydrogel [125] (Reproduced with permission). (**iiia**) Formation of H-CD monomers and their aggregates (the disulphide bond in dithiosalicylic acid molecular is highlighted with yellow). (**iiib**) Photographs of the H-CD’s two-switch-mode luminescence principle. (**iiic**) Fluorescence principle and proposed structure of H-CD’s core and surface [129] (Reproduced with permission).

A multi-responsive and hybrid white-light-emitting hydrogel network was developed recently by incorporating Eu^3+^, Tb^3+^, and CD into polyacrylamide/poly (acrylic acid) hydrogel network. In the network, luminescent lanthanide ions were incorporated with coordination bonding, whereas CDs interacted with radical copolymerization. The white luminescence was achieved by modifying the ratio of blue-light-emitting CD to green- and red-light emitting lanthanide ions. The photoluminescence study revealed that the mechanism involves energy-transfer from Tb^3+^ to Eu^3+^ and CDs, as shown in Figure 4i,ii. The reaction between lanthanide and CDs resulted in the responsiveness of white-light-emitting hydrogel to various stimuli such as pH, vapours, metal ions, and temperature. Additionally, the hybrid network showed thermochromism of green-to-red emission ratios in the 20–70 °C temperature range and a fracture strain stretchability of 400% [125]. Recently, a white-light-emitting hydrogel developed by mixing lanthanide (Eu^3+^), cytidine, fluorescein isothiocyanate, and CDs was found sensitive to pH, Fe^3+^ and temperature [126]. Further, a smart dual-mode sensitive nano-probe based on CDs-Tb-TMPDPA was developed in which Tb^3+^ and CDs were sensitive to temperature and photothermal, respectively, and 4-(2,4,6-trimethoxyphenyl)-pyridine-2,6-dicarboxylic acid (TMPDPA) acted as a two-photon ligand. The developed nano-probe can be ideal for achieving real-time temperature and optical heating simultaneously [127]. Wang and co-workers prepared CDs by polymerizing reaction of citric acid, hyaluronic acid polymers, and ethylenediamine that were smartly utilized as gatekeepers to prevent premature drug release, for targeted drug delivery to tumour cells, IR thermal imaging, and thermo-chemotherapy [128]. The possibility of employing CDs as reversible two switch-mode luminescence ink for advanced anti-counterfeiting and dual encryption was explored. For which CDs were synthesized from melamine and dithiosalicylic acid using a solvothermal method that exhibited blue fluorescence. When mixed with water, the hydrophobic CDs resulted in aggregation leading to red fluorescence as shown in Figure 4iiia–iiic. This phenomenon was attributed to the constraints in the intramolecular rotation of disulphide bonds and the interaction of π-π stacking in aggregated structures [129].

### 5.6. Phase Sensitive

**Figure 5:**
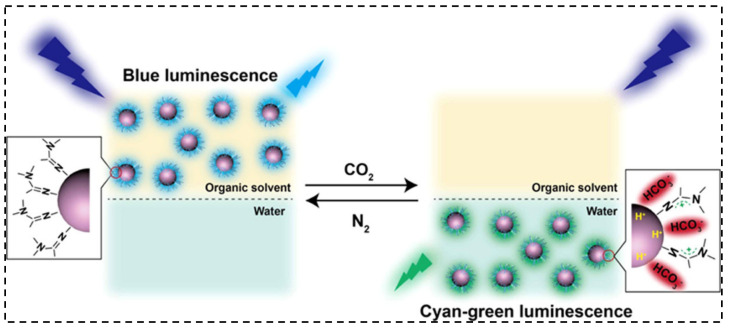
The reversible phase transfer of the amidine-modified CDs and the reversible luminescence change between blue and cyan-green luminescence change in the presence of CO_2_ and N_2_ [131] (Reproduced with permission).

Photoluminescence-functionalized phase change materials (PCMs) have also attracted attention because of their potential application as smart materials. The PCMs with the property of capturing excess energy and releasing it via phase transition are primarily used in thermal energy management. In a work, a novel MOF-based photoluminescence-functionalized PCMs was developed by incorporating stearic acid (SA) and carbon quantum dots (CQDs) that served as a fluorescent guest and thermal energy guest, respectively, into the Cr-MIL-101-NH_2_ framework. The nanocomposite possessed remarkable thermal stability, durability, and shape-stabilized ability and prevented conventional aggregation-induced quenching [130]. Smart CDs were also designed wherein CO_2_ and N_2_ bubbling led to both reversible phase transfer between the organic and aqueous phase of the CDs and simultaneously resulted in reversible luminescence change from blue to cyan-green as shown in Figure 5. This phenomenon has been ascribed to the modification of surface chemistry and different emission states that is activated by the CO_2_/N_2_ bubbling [131].

### 5.7. Solvent Sensitive

The optical response of CD’s is also dependent on the solvent polarity. Li et al. developed green- and blue-emitting carbon quantum dots (CQDs) from oxalic acid, citric acid, and urea as precursors. The CQDs exhibited solvent selectivity with the green CQDs soluble in the ethanol, and the blue-CQDs soluble in water. Such an interesting phenomenon could be because of the structural difference between the two CQDs. The work also demonstrated the reversibility of colour by changing the hydrogen bonding, which is more straightforward than the pressure application for colour reversibility [132]. Different hydrochromic CDs were also fabricated from brown sugar following a simple carbonization method that displayed variation in light emission in the presence of water. The synthesized CDs displayed a “structure-hydrochromic property” relationship wherein the CDs with the superior pi character had hydrochromism. The CDs exhibited red shift in the presence of water in aprotic solvents, whereas, in protic solvents, the emission intensity was increased. Therefore, this property of the CD was employed to probe water contamination in different solvents [133]. Different solvents have different emission wavelengths. Solvent properties such as dehydrating ability, solubility, boiling point, polarity, and (non-) proton influence the particle size and surface state of the synthesized CD [134].

## 6. CDs in Optical-Based Analytical

As evident from the previous sections, the CD’s inherent optical properties are remarkably altered in response to one or more external stimuli. Therefore, exploiting the CD’s role in a variety of smart applications has become of intense interest.

### 6.1. Photoluminescence

One of the most fascinating properties of CDs is the photoluminescence or fluorescence. The fluorescence phenomenon occurs when the electronically excited fluorophore relaxes from the singlet state to the ground state. Many recent studies have shown noteworthy advances in the field of fluorescence. CDs are frequently used as fluorescent probes for the detection of bioanalytes, small molecules, and ions.

Most fluorescence-based detection methods use turn-on, and turn-off modes wherein the appearance or disappearance of the colour is sometimes difficult to differentiate. Therefore, to increase the visibility in biosensors, a combination of two fluorophores is often used, wherein only one of the fluorophores is sensitive to the analyte, and the other one is inert. Recently, using CD and rhodamine 6G (Rh6G) fluorophore, a ratiometric fluorescence-based experiment was designed for the detection of glucose in both the aqueous phase and on solid-state hydrogel film by employing glucose oxidase (GOx) and horseradish peroxidase (HRP) enzyme. The developed system resulted in the quenching of the blue-colour emitting CD, whereas the green-colour emitting Rh6G had no response and, therefore, with increasing concentrations of glucose in the CD/Rh6G/GOx/HRP, the colour of the fluorescence changed from blue to green. For the solid film detection, the CD/Rh6G/GOx/HRP was immobilized in a hydrogel film developed by UV curing of 70:30 (*w*/*w*) mixture of acrylic acid and diacrylate poly (ethylene glycol). The experimental results indicated that in contrast to the aqueous phase, the solid-state platform was much more selective to glucose, had high stability, and could be reused [135]. A ratiometric CD-based fluorescent probe for the detection of lead (Pb^2+^) and pyrophosphate (PPi) was synthesised. The CDs displayed a three-state “pink-cyan-pink” emission upon the addition of Pb^2+^ and PPi. The Pb^2+^ formed a coordination complex with the amine and porphyrin group on the surface of the CD that resulted in the quenching of the fluorescence. Interestingly, upon the addition of PPi, the CD’s fluorescence was recovered, attributed to the removal of Pb^2+^ from the CD’s surface. Based on this, a fast and straightforward paper-based platform for the detection of Pb^2+^ and PPi was designed [136].

In recent times, CDs have attracted attention for their potential applications in the field of optoelectronics. To this end, various designed strategy has been developed using CD as fluorescence signal probe. Doping of the CD as an efficient strategy has many reports on the field. For example, nitrogen and sulphur co-doped (N, S) CDs using *O*-Phenylenediamine (OPD) and sulphamide were developed that exhibited excitation-independent properties with bright red fluorescence. A mixture of N, S-CDs along with blue- and green-emitting CDs was used to develop white light-emitting polyvinylpyrrolidone (PVP) film that was subsequently layered on a UV-LED chip to design white light-emitting diodes (WLEDs). The fluorescence intensity of the developed WLEDs remained stable for 20 days and at varying temperatures. The polymer chains in the CDs generated repulsive forces that resulted in the stability of the WLEDs at different temperatures. Further, the fluorescence intensity of the N, S-CDs was quenched in the presence of water, and therefore was employed for the sensitive detection of water in organic solvents [137]. Further, carbon dot/hydrogel composites have also been used to obtain effective sensing platforms. Using cellulose nanofibrils (CNFs) and CDs, a novel fluorescent hydrogel was developed that displayed a high QY of 23.6%. The CNF’s natural skeleton and the amide, hydroxyl, and carboxyl groups on the hydrogel surface allowed the accumulation and adsorption of heavy metal ions. Further, the hydrogel’s three-dimensional network generated channels for the diffusion of the ions from the outside to the inside of the adsorption sites, with high adsorption capacity for Fe^3+^, Ba^2+^, Pb^2+^, and Cu^2+^. Additionally, the hydrogel’s fluorescence was rapidly quenched in the presence of Fe^2+^, and therefore, was applied for the visual optical detection of the ion. The quenching phenomenon is attributed to the constraints of the hydrogel’s fluorescence excitation as a result of the adsorption of the Fe^3+^ [138]. In another interesting study, an antibacterial wound dressing was developed from a nanocolloidal hydrogel composed of CD/cellulose nanocrystal hybrid nanoparticles and gelatin. The hydrogel displayed high Fe^3+^ ion absorption capacity, limiting the accessibility of these ions required for the pathogenic bacteria’s growth at the infection site. As such, the hydrogel absorbed Fe^3+^ ion interacted with the surface functional group of the CD that resulted in the quenching of the hydrogel’s photoluminescence [139].

The CDs are also attracting interest for their application as nanothermometers due to their property to undergo alterations in their fluorescence property in response to temperature stimuli. Mohammed and co-workers reported boron and nitrogen co-doped (B, C) CDs that exhibited variation in the fluorescence intensity at different temperatures (0–90 °C) with a thermo-sensitivity of 1.8% °C^−1^. Therefore, it can be employed as efficient nanothermometers. The temperature did not influence the CD’s surface fluorescent structure as the fluorescence intensity was easily reversed and restored when the temperature was increased and subsequently decreased [140].

The optical-fibre technique in biosensing applications in recent times is highly sought-after. In one such development, a novel optical-fibre glucose biosensor exhibiting high sensitivity and continuous online detection of glucose has been developed. The end face of the optical fibre was immobilized following dip-coating method using carbon quantum dots (CQDs)-glucose oxidase (GOD)-cellulose acetate (CA) film, allowing visualization of real-time glucose fluctuations in various concentration ranges including micro-to nano-mole levels [141].

Surface modification of CDs is emerged as suitable strategy to facilitate its application in optical-based detection. In a study, a novel thymine functionalized carbon dot (CDs-Thy) was designed to detect Hg^2+^. In the presence of Hg^2+^, the thymine moieties on the surface of carbon dot could form T-Hg^2+^-T structures, allowing photoinduced electron transfer to occur from the excited CDs-Thy to the vacant d orbital of Hg^2+^. This event resulted in the quenching of the CDs-Thy. In the proposed method, the Hg^2+^ was detected in the linear concentration range of 0–1.0 μmol L^−1^ and a limit of detection (LOD) 3.5 × 10^−8^ mol L^−1^ [142]. In another study, for the detection of hypochlorite (ClO^−^), a novel dual emission ratiometric probe (FH-GA-CQDs) was synthesized by covalently conjugating fluorescein (FH) derivative (green-colour emission) onto the surface of CDs (CQDs) (blue-colour emission) via the GA linker. In the presence of ClO^−^, the xanthene ring of the FH unit opens via oxidative induction, which allows the FRET process to occur from CQD to the FH units resulting in the decrease of the fluorescence intensity of CQD and increase of the fluorescence intensity of FH units. By using this method, ClO^−^ as low as 93 nM (LOD) could be detected [143]. In a recent past, a FRET-based system was developed using CDs as energy donor and gold nanorods (Au NRs) as energy acceptor. By using cysteamine as a bridge, the nanomaterials were covalently connected, hindering the FRET process. Upon the addition of Pb^2+^, cysteamine interacted with the ion resulting in activation of the fluorescence signal as the FRET process was disturbed. The Pb^2+^ could be detected through this sensor system with high selectivity and sensitivity with 0.05 µM as LOD [30]. Table 3. Illustrates the various other reports of carbon dot as smart material in PL sensing.

#### Dual-Mode Detection Systems

CD-based dual-function optical systems are expected to offer confirmed detection along with increasing sensitivity for the target. A dual-mode nitrite (NO_2_^−^) detection method using N-doped carbon dot was synthesized using phenosafranine and citric acid through hydrothermal treatment. The surface-oriented amino group of the N-CD coordinated with the NO_2_^−^ resulting in aggregation of the carbon nanoparticles. This event caused static fluorescence quenching of the orange emitting CD. Additionally, with increasing concentrations of NO_2_^−^, the colour of the N-CD solution changed ratiometrically from red to purple. The work has potential to detect NO_2_^−^ in A549 cells [161]. In a similar study, a smart detection of 2-nitrophenol (2-NP) and 4-nitrophenol (4-NP) using a carbon dot synthesized from EDTA having dual-readout property was demonstrated. Upon the addition of both 2-NP and 4-NP to the CD solution, the fluorescence intensity dropped significantly. The fluorescence quenching of the CD was attributed to the inner filter effect. Besides, the system having CD and 4-NP resulted in the splitting of the corresponding CD peak into two, along with the fluorescence quenching. At the same time, the CD solution turned colourless to yellow in the presence of both 2-NP and 4-NP [162].

Previously, a red-emitting CD via a hydrothermal route using p-phenylenediamine and phosphoric acid as precursors was synthesised. The CD was employed in an “on–off-on” dual-mode sensing platform for the sequential detection of chromium [Cr(VI)] and cysteine (Cys). The fluorescence of the red-emitting CD was dramatically quenched upon its interaction with Cr(VI) owing to the binding of the latter to the surface functional groups that resulted in charge transfer from the excited state CD to the Cr(VI). Addition of Cys to the CD@Cr(VI) complex restored the fluorescence of the CD, due to the formation of a more stable Cr(VI)@Cys complex. Concurrently, the colour of the CD solution also changed from red to purple to yellow. By integrating the merits of the phenomenon, the nanoprobe was used in cellular imaging and the construction of AND logic gate [29]. Subsequently, a strategy was used to simultaneously detect Cu^2+^ and Al^3+^ using a dual emissive CD synthesized from red tea through a solvothermal treatment. The CD exhibited strong and weak emission peaks corresponding to the red and blue regions, respectively, at an excitation of 410 nm. In the presence of Cu^2+^, the red emission peak quenched utterly, whereas the intensity of the blue emission peak increased and resulted in a red-shift in the presence of Al^3+^. This phenomenon is attributed to aggregation-induced emission quenching (ACQ) and aggregation-induced emission enhancement (AIEE) of the CD upon its interaction with Cu^2+^ and Al^3+^, respectively. Additionally, under the UV light, the fluorescence colour of the CD solution changed from red to orange and then yellow to green in the presence of Al^3+^, which was visible to the naked eye [163]. In a recent work, pH-sensitive gadolinium (III) (Gd^3+^)-doped CDs was synthesised using citric acid, GdCl_3_, and urea via a solvothermal route. The CDs displayed excitation-independent bright red fluorescence and a high T1 relaxivity of ∼16.0 mM^−1^ s^−1^. The CDs showed fascinating pH-dependent responses in both fluorescence (FL) and magnetic resonance signals. At acidic pH, the fluorescence of Gd^3+^-doped CD quenched and exhibited increased brightness of the MR image. However, at alkaline pH, the Gd^3+^-doped CD displayed bright red fluorescence, and the response of the MR changed from bright to dark. They found that the pH-dependent responses vary reversibly when the pH is switched repeatedly from 10.0 to 1.0. Additionally, based on the Gd^3+^-doped CD, they also developed an FL/MR dual-readout logic gates, wherein H^+^, OH^−^, Cu^2+^ acted as input as they stimulate the fluorescence and MR response in a switched manner. Based on this phenomenon, the Gd^3+^-doped CD was employed in bioimaging [164]. Table 4 lists some of the other reported CDs with dual-functionality.

### 6.2. Chemiluminescence

Chemiluminescence (CL), a type of luminescence, is the phenomenon of emission of light resulting from a redox chemical reaction between the reagents. Typically, the reaction produces electronically excited intermediates or products, which, when relaxes to the ground state, release energy as photons of light. The chemiluminescence method is a widely used optical detection method due to its distinctive features such as high sensitivity, no requirement of an external light source for excitation, simple instrumentation, and fast response time. Luminol, potassium permanganate, lucigenin, tris(2,2-bipyridine) ruthenium (II), and peroxyoxalate are the most commonly used classical CL reagents in analytical applications. However, these classical CL reagents are expensive, most are poisonous, have low selectivity, and, most importantly, generate weak CL intensity, which is a major drawback. HCO_4_^−^ and HSO_3_^−^ are some of the green and cheaper CL reagents that are frequently used in combination with various oxidants in CL reaction systems. Nevertheless, these systems also produce weak or ultraweak CL intensity. Therefore, in the past few years, there has been tremendous research on incorporating nanomaterials in CL systems in order to enhance the intensity of CDs, a less/nontoxic and inexpensive fluorophore, have emerged as a suitable candidate for increasing the CL reaction system’s applicability. CDs have been growingly employed as enhancers, catalysts, energy acceptors, or emitter in CL studies. In some instances, the carbon nanomaterial is also found to play multiple roles in a single CL reaction system. Hence, it is important to explore the stimuli of CD as well as its application in a CL reaction system. Carbon nitride quantum dots (CNQD) derived from sodium citrate and urea using a solvothermal method found to enhance the CL emission intensity of hydrogen peroxide (H_2_O_2_) and hydrosulphite (HSO_3_^−^) reaction system. It was confirmed that in the presence of the CNQD, there was an enhancement of the singlet oxygen (^1^O_2_), superoxide radical (O_2_^−^), and sulphite anionic radical (**^.^**SO_3_^−^), which might be responsible for the increased chemiluminescence intensity of the CNQD-NaHSO_3_-H_2_O_2_ reaction system. The study further indicated that the main emitters are SO_2_*, ^1^O_2_, (O_2_)_2_*, and the excited state CNQD. The CL spectra specified the formation of excited-state CNQD as a result of accepting energy from SO_2_*, ^1^O_2_, (O_2_)_2_* followed by the subsequent release of the CL. Additionally, it is assumed that the CNQD transferred electrons to the OH radical and O_2_ that ultimately increased the number of holes in CNQD. This event stimulated the electron-hole annihilation process, which further resulted in enhanced chemiluminescence. Interestingly, the order in which reagents were mixed in the system was found to influence the CL intensity. The presence of ascorbic acid in the reaction system was found to have an inhibitory effect on the CL phenomenon. Therefore, the CNQD-NaHSO_3_-H_2_O_2_ system was employed to determine ascorbic acid with an LOD of 8.0 × 10^−8^ mol L^−1^ [171].

In a recent effort, K_2_Cr_2_O_7_ was used as an oxidant that oxidized NaHSO_3_ resulting in the generation of ultraweak chemiluminescence. The synthesized carbon dot (MFCD) acted as an energy acceptor from the excited sulphur dioxide (SO_2_*), followed by returning to the ground state with enhanced CL intensity. In this case, OH and O_2_^−^ were the primary intermediate radicals involved in chemiluminescence. Additionally, these oxygen radicals might be responsible for the generation of electron-injected (MFCD^−^) and hole-injected (MFCD^+^) CDs that gave rise to MFCD* by radiative electron-hole annihilation, which further enhanced the CL intensity. Apart from that, it is often observed that the chemiluminescence spectrum of carbon dot is red-shifted compared to its fluorescence spectrum. The shifting to higher wavelength is presumed to be because of the smaller band gap energy of the surface state compared than the core state responsible for fluorescence. Moreover, in the presence of iodide, the chemiluminescence was amplified, and hence the system was used for probing iodide [172].

Amjadi et al. demonstrated that the chemiluminescence intensity of HCO_3_^−^·H_2_O_2_ was enhanced to 70-fold in the presence of both silicon-doped CD (Si-CD) and CTAB with a response time of 10 s. This effect has been attributed to the synergistic interaction of Si-CD and the CTAB. The cationic surfactant most probably formed micellar structures on the surface of the carbon dot that attracted the oxygen radicals (OH and O_2_^−^). Later, these radicals expedited the process of production of electron-injected and hole-injected CD resulting in enhanced chemiluminescence. The micellar structure also acted as a shield for the excited-state CD to decrease non-radiative deactivation. Based on this phenomenon, a simple and sensitive method was developed for the determination of dopamine, adrenaline, and noradrenaline in biological samples [173].

The presence of nitrogen in CDs have a remarkable effect on the CL signal. Hallaj et al. tested different types of luminescent CDs among which carbon nitride quantum dots (CNQDs) were found to dramatically boost the chemiluminescence intensity of the reaction system by a factor of about 75. The nitrogen-containing functional groups on the CNQD stimulated the decomposition of H_2_O_2_ into oxygen radicals and also caused chelation of Cu^2+^ ions that resulted in generation of redox-active species for reducing H_2_O_2_ with higher degree. The chemiluminescent system was found to have a linear relationship with increasing concentrations of H_2_O_2_ and therefore was employed for the determination of both H_2_O_2_ (LOD: 10 nM) and glucose (LOD: 100 nM) [174]. Transition metal ions such as Fe^2+^ and Cu^2+^ have also been studied to a great extent for enhancing CD-based chemiluminescence. These metal ions can decompose H_2_O_2_ to hydroxyl radical (OH) via the Fenton mechanism. Next, the **^.^**OH cause the generation of electrons and holes in the valence and conduction band of CD resulting in the enhancement of chemiluminescent signal. Based on this phenomenon, Shah et al. developed a method for the determination of Fe^2+^ using a N-doped CD synthesized from histidine and H_2_O_2_ as a coreactant [175].

To further increase the versatility, Duan et al. fabricated copper-doped CDs (Cu-CDs) using citric acid via solid-phase synthesis strategy that exhibited excellent peroxidase-like activity. The incorporation of Cu^2+^ to the CDs as a dopant enhanced the electronic properties and surface reactivity. The developed Cu-CD was employed as a chemiluminescent probe for the detection of glucose that displayed a detection limit of 0.32 μM [38]. There are several reports of CDs as a smart material in CL system (Table 5).

### 6.3. Electrochemiluminescence

Electrochemiluminescence (ECL), a combination of electrochemistry and luminescence, is the emission of light by an excited-state species originating from high-energy electron transfer reactions at electrodes. Generally, the ECL phenomenon occurs by two pathways: The annihilation pathway and the coreactant pathway [182]. In the annihilation pathway, following the applied potential, oxidized and reduced species are formed at the electrode’s surface, which subsequently generates the emissive excited state. The coreactant pathway, in contrast, contains an electroactive luminophore as well as a coreactant that is responsible for ECL emission. Compared to the annihilation pathway, the coreactant pathway has more advantages with stronger ECL emission and is suitable for both aqueous and nonaqueous electrolytes. As a new class of the carbon nanomaterial, the CDs due to its high QY, high sensitivity, and low background signal have shown promising applications as an ECL luminophore and a coreactant for the detection of numerous analytes.

Solid ECL platform for the detection of Cu^2+^ ion using phosphorus-doped carbon quantum dots (P-CQDs) with H_2_O_2_ as a coreactant has been reported. The corresponding QY and the ECL intensity of the P-CQD were 2- fold and 4-fold, higher than the undoped CQD. The increased ECL intensity is because of the phosphorus doping that created emissive traps, and high electron transfer reaction between the electrogenerated P-CQD intermediate (P-CQD^−^) and the reduced H_2_O_2_. The OH radical injects hole into the HOMO level of P-CQD^−^ giving rise to the excited P-CQD (P-CQD*) that emits light. Accordingly, the method was employed to detect Cu^2+^, a Fenton reagent, that results in the generation of more OH radicals from H_2_O_2_, resulting in enhanced ECL intensity. The developed P-CQD could detect as low as 0.27 nM Cu^2+^ [183]. A sandwich-type novel ECL immunosensor was also fabricated to determine carcinoembryonic antigen (CEA) by using perylenetetracarboxylic acid (PTCA) and CQDs as dual luminophore, graphene as nanocarrier and S_2_O_8_^2^^−^ as a coreactant, which exhibited good performance for the detection of CEA in human serum samples. The secondary antibody used to capture the CEA was immobilized on the ECL nanomaterial. Because of the synergistic effect of the luminophores, the sensor displayed a remarkable increase in the ECL intensity. The coreactant reacted with the reduced species of PTCA (PTCA^−^) and CQDs (CQDs^−^) to form the corresponding excited species, which emitted light. The proposed method could detect CEA as low as 0.00026 fg mL^−1^ [184].

Based on the fact that Hg^2+^ can form T-Hg^2+^-T complex with two DNA thymine (T) bases, a novel ECL sensor was developed to detect Hg^2+^ by using GQDs-DNA-AuNP as the ECL nanoprobe and poly(5-formylindole)/reduced graphene oxide (P5FIn/erGO) as nanocomposite. A thymine rich ssDNA immobilized on the P5FIn/erGO modified electrode formed the T-Hg^2+^-T complex in the presence of both Hg^2+^ and the ECL nanoprobe. With increasing Hg^2+^ concentration, the ECL intensity of GQD also increased, and by using this relation, the ECL sensor developed that could detect as low as 2.48 pM Hg^2+^ with good stability, selectivity, and reproducibility. The combination of AuNP, GQD, and P5FIn/erGO in the sensor accelerated the electron transfer rate, an increased loading capacity of ssDNA, and high ECL intensity, ultimately resulting in a lower detection limit [185].

While carbon nitride quantum dots (GCN QDs) have several advantages, the low ECL efficiency is often a drawback in the analytical applications. To improve the efficiency in ECL assays, a novel luminophore was developed by doping GCN QDs with sulphur (S-GCN QD) that generated element vacancy and also modified the surface state. By using the surface plasmon coupling ECL (SPC-ECL) mode, wherein the ECL intensity can be amplified by surface plasmon coupling effect of AuNP, the S-GCN QDs were used to develop a sandwiched ECL sensor to detect K-RAS gene which is a crucial cancer biomarker [186]. In a similar study, a novel nitrogen doped hydrazide conjugated CDs was developed that had high quantum efficiency with emission at low excitation potential. The proposed carbon dot was employed to distinguish cancer cells from the normal cells on the basis of more hydrogen peroxide secretion from the former [187]. In Table 6 the various other reports of CDs acting as a smart material in ECL systems is shown.

## 7. Conclusions and Future Perspectives

This review witnesses rapid progress on CD’s research, primarily on its smart material properties and their exploitations for developing various optical-based detection systems. Several stimuli-responsive properties of the CDs have been identified, which concurrently inspired to develop new strategies for the detection of a wide array of analytes with special efforts seen in environmental monitoring and healthcare applications such as the detection of toxic heavy metals, chemicals, and compounds/cells of clinical importance. The CD’s optoelectronic and surface chemical properties are seen as guiding factors for various sensitive stimuli-responsive signals, which led to acquire highly sensitive optical behaviours such as photoluminescence and chemiluminescence, and electrochemiluminescence in the detection processes. The optical response-based smart material properties of CDs are greatly influenced by the synthesis route of these 0-dimensional carbon nanomaterials. Consequently, the surface passivation and allied hybrid synthesis techniques of the CDs are emerged to gain their functional benefits. However, despite the progress made, the smart material research on CDs is still in its infant stage compared to the other carbon nanomaterials. There is enough scope to enhance their performance further through engineering the material properties to develop CDs for generating custom-made optical signals for various sensing and imaging applications. A simple and straightforward synthesis strategy is important for scaling up their production with uniform size and intended applications for commercial success. Secondly, elucidation of detailed mechanisms on the external stimuli led-change or generation of the optical properties will help developing CDs through rational design. It is undeniable that the CD’s attractive optical properties will bring about more exciting applications as a smart material in the coming days.

## Figures and Tables

**Table 1:** Synthesis of carbon dots (CDs) following top-down and bottom-up approach.

Precursors	Synthesis Route/Temp/Time	Types of CDs	Size(nm)/QY (%)		Ref
QR, NaOH	Ultrasonic	CDs	2.99/27.7		[11]
	RT/6 h				
CA, PEG	Ultrasonic	CDs	2.38/NA		[12]
	RT/1 h				
Soybean	Ultrasonic	NBDs	24/16.7		[13]
	2 h				
Graphite, ethanol	Laser ablation	CDs	F_s_	1.4/NA	[14]
	800 nm, 150 fs		2F_s_	2/NA	
			F_d_-2	1.1/NA	
			F_d_-10	1.5/NA	
DEASA, H_3_PO_4_	Hydrothermal	CDs	4.69/19.4		[15]
	200 °C/2 h				
Jeera	Hydrothermal	CDs	6–9/5.33		[16]
	250 °C/6 h				
Tobacco	Hydrothermal	CDs	2.14 ± 0.3/27.9		[17]
	300 °C/3 h				
*Cryptococcus*	Hydrothermal	CDs	4–9/14.13		[18]
	160 °C/1 h				
TMA	Hydrothermal	CDs	4.3/13.4		[19]
	260 °C/12 h				
*Agaricus bisporus*	Hydrothermal	CDs	NA/4.2		[20]
	160 °C/12 h				
Chitosan, AA	Hydrothermal	CQDs	5/NA		[21]
	180 °C/12 h				
CA, EA	Hydrothermal	CDs	4/NA		[22]
	180 °C/8 h				
Folic acid, Glucose	Hydrothermal210 °C/12 h	CDs1	1.6/23.5		[23]
		CDs2	2.6/42.8		
Xylose, H_3_PO_4,_	Microwave	CDs1	6.80/73.6		[24]
m-PD	220 °C/10 min				
Xylose, H_3_PO_4,_	Microwave	CDs2	7.23/56.1		
m-PD	200 °C/10 min				
Xylose, H_3_PO_4,_	Microwave	CDs3	6.80/40.9		
m-PD	180 °C/10 min				
Xylose, m-PD	Microwave	CDs4	9.88/65.3		
HNO_3_	220 °C/10 min				
Xylose, m-PD	Microwave	CDs5	8.83/49.5		
CH_3_COOH	220 °C/10 min				
Xylose, m-PD	Microwave	CDs6	6.87/42.8		
	220 °C/10 min				
Xylose, m-PD, Na_3_PO_4_	Microwave	CDs7	16.37/8		
	220 °C/10 min				
Xylose, m-PD, NaOH	Microwave	CDs8	10.70/6.8		
	220 °C/10 min				
CA, EDA	Microwave	CDs	4/24		[25]
	720 W/2 min				
CA, urea	Microwave	CDs	NA/NA		[26]
	750 W/5 min				
Anthracite coal, H_2_SO_4_, HNO_3_	Pyrolysis in oil bath	CDs	3.5/NA		[27]
	120 °C/24 h				
CA, Glu, Asp, lysine	Thermal pyrolysis	CDs	4/8.8		[28]
	200 °C/30 min				
CA, acrylamide	Solvothermal	CDs	9.5/NA		[29]
	200 °C/4 h				
CA	Oven	CDs	3/NA		[30]
	200 °C/1h				

DEASA: 4-(diethylamino) salicylaldehyde; CA: Citric acid; EA: Ethanolamine; AA: Acetic acid; SC: Sodium citrate; QR: quinaldine red; RT: room temperature; PEG: poly(ethylene glycol); NBDs: nano biomass dots; F_s_: single-pulse ablation (F_single_ = 50 J·cm^−2^); 2F_s_: single-pulse ablation (2F_single_); F_d_-2: double-pulse ablation (τ_delay_ = 2ps); F_d_-10: double-pulse ablation (τ_delay_ = 10 ps); EDA: ethylene diamine; TMA: Trimellitic acid; m-PD: m-phenylenediamine; NA: Not Available.

**Table 2:** Synthesis of heteroatoms doped CDs following top-down and bottom-up approach.

Precursors	Synthesis Route/Temp/Time	Types of CDs	Size (nm)/QY(%)		Ref	
Cryomilled graphite, DMF	Laser ablation	N-CDs	3/4.05		[40]	
	800 °C/3 h					
2-aminopyrimidine-5-boronic acid	Laser ablation	N, B-CD	3/58		[41]	
	170 mW/1014 Wcm^−1^					
Graphite rods and ammonia hydroxide	Electrochemical and ultrasonic	N-CDs	3–5/NA		[42]	
	80 °C/3 h					
L-tryptophan, chlorhexidine acetate	Hydrothermal	N-CDs	~4.0/NA		[43]	
	200 °C/12 h					
*P. acidus*, aq. ammonia	Hydrothermal	N-CDs	5/12.5		[44]	
	200 °C/12 h					
PVP	Hydrothermal	N-CDs	6.5/6		[45]	
	200 °C/6 h					
EDA, CuCl_2_.2H_2_O	Hydrothermal	Cu-CDs	1.8/7.8		[46]	
	180 °C/10 h					
Aphen, CA	Hydrothermal	AC-CDs	25/52		[47]	
	200 °C/7 h					
OPD, ABPA	Hydrothermal	B,N-CDs	4.09/8.56		[48]	
	160 °C/6 h					
Mn(III)(C_5_H_7_O_2_)_3_	Hydrothermal	MnO_x_-CDs	5.65 ± 0.30/11.3		[49]	
	200 °C/12 h					
Sucrose, nitrobenzene, nitrosobenzene	Hydrothermal	CD-NO, and CD-NO_2_	gCD	11/21	[50]	
	180°C/12 h		rCD	13/18		
CA, EDA	Microwave	N-CDs	~5/95, 11		[51]	
CA, EDA, sodium borate	300 W/10 min	B-CDs	~5/63, 9			
CA, EDA, K_2_PO_4_		P-CDs	~5/63, 6			
p-PDA, EDA	Microwave	N-CDs	4.8/14		[52]	
	500 W/20 min					
TSCDH, Urea, DMF	Solvothermal	N-CD_11_	4.5/21.6		[53]	
TSCDH, Urea, DMAC	160 °C/4 h	N-CD_12_	4.5/18.7			
TSCDH, Urea, DEF		N-CD_21_	4.5/17.6			
H_2_O_2_, ethanol, NH_3_	Solvothermal	N-CDs	2.15/56.1		[54]	
	180 °C/NA					
CA, PD	Solvothermal	Y-CDs	7.2/24		[55]	
	170 °C/4 h					
CA and DAN	Solvothermal	HCP-DB-CDs	2.4/70 ± 10		[56]	
	160 °C/6 h					
PAA, CuN, HH, (NH_4_)_2_S_2_O_8_	Carbonization/polymerization and pyrolyzation	Cu-CDs	2.8/36		[57]	
	Stirring/24 h and 400 °C/90 min					
Willow Catkin, Urea and H_2_SO_4_	Combustion	N,S-CDs	7.3/14.3		[58]	
Na_2_[Cu(EDTA)] and Ascorbic acid	Thermolysis	Cu-CDs	3.48/9.8		[59]	
	250 °C/2 h					

PAA: Poly(acrylic acid); CA: Citric acid; MA: Melamine; DTSA: Dithiosalicylic acid; EDA: Ethylenediamine; TSCDH: Trisodium citrate dihydrate, DMF: dimethylformamide, DMAC: dimethylacetamide; DEF: dimethylformamide; EA: Ethanolamine; Glu: Glutamic acid; Asp: Aspartic acid; AA: Acetic acid; TNP: 3,4,9,10-tetranitroperylene; PEI: poly(ethyleneimine); p-PDA: p-phenylenediamine; OPD: O-phenylenediamine; ABPA: 3-aminophenylboronic acid; PD: 2,3-phenazinediamine; Aphen: 5-amino-1,10-phenanthroline; Ru-Aphen: 5-amino-1,10-phenanthroline ruthenium (II) complex; AC: ammonium citrate; SBH: sodium borohydride; DAN: diaminonaphthalene; CuN: Copper nitrate; HH: Hydrazine hydrate; DMF: dimethylformamide; NA: Not Available.

**Table 3:** Carbon dot-based photoluminescence detection.

Precursor and Synthesis Route	Size (QY)	Application	Ref
Citric acid, urea, and thiourea	10 nm (19.2%)	Mercury (II) and iodide detection	[144]
Microwave-assisted			
Succinic acid and glycerol	2.3 nm (11%) (Blue-CD)	Fe^2+^, H_2_O_2_ detection and bioimaging	[145]
Hydrothermal	4.6 nm (7%) (Green-CD)		
L-glutamate	2 nm–4 nm (34%)	*Plasmodium falciparum* glutamate dehydrogenase detection	[146]
Pyrolysis			
Citric acid and ethylenediamine	3.6 nm (NA)	Detection of glucose	[147]
Microwave-assisted hydrothermal			
Citric acid, formamide, and ethanol (E-CD)	4.5 nm (15.81%—water, 22.43%—DMSO, 25.80%—DMF, 19.42%—methanol) (E-CD)	Fluorescent pH sensor	[148]
Citric acid, formamide (N-CD)			
Solvothermal	5.5 nm (NA) (N-CD)		
Sodium citrate and urea	3.52 nm (67%)	Mercury ion detection in living cells and visualization of latent fingerprints	[149]
Solvothermal			
Citric acid and ethylenediamine	2 nm (NA)	Doxycycline and MnO_4_^-^ detection	[150]
Hydrothermal			
Leaf extract of *Bougainvillea*	10.7 nm (~41%)	Bioimaging, detection of Cu (II), and as red-emitting fluorescent ink	[151]
Microwave-assisted			
Pricky pear cactus	5.6 nm (12.7%)	Arsenic (III) and hypochlorite ion detection in drinking water	[152]
Hydrothermal			
Citric acid and glycine	2.8 nm (78%)	Detection of chromium (VI)	[153]
Hydrothermal			
p-phenylenediamine	3.8 nm (15%)	Detection of pH and Fe^3+^	[154]
Microwave-assisted			
Glycerol and cysteine	1–6 nm (3.5%)	Detection of Hg(II)	[155]
Microwave-assisted			
Sodium lignosulphonate and p-phenylenediamine	2.02 nm (11.25%—ethanol, 13.77%—n-propanol, 11.66%—isopropanol, 15.07%—DMF, 14.29%—DMA), 5.12% (water), 5.58% (acetic acid), 5.77% (propionic acid)	Detection of Fe(III), Ag(I) and as a solvatochromic probe	[156]
Solvothermal			
Glutathione, sodium citrate, (blue-CDs) and 1,2,4-triaminobenzene (yellow-CDs)	4.0 nm (Blue-CD)	Detection of Fe^3+^ and PPi	[157]
	2.4 nm (Yellow-CD)		
	(N A)		
Hydrothermal (blue-CDs), Solvothermal (yellow CDs)			
Cellulose-based willow catkin biowaste	7.3 nm (13.3%)	Detection of Fe^3+^ and bioimaging	[58]
Combustion treatment			
Melamine and dithiosalicylic sacid	6.5 nm (5.96%)	Two-switch-mode luminescence ink	[129]
Solvothermal			
Glucose and HAuCl_4_	10 nm (0.15%)	Detection of Pb^2+^	[158]
Microwave-assisted			
Alizarine carmine	2.37 ± 0.23 nm (6.3%)	Detection of glutathione and cancer cells.	[159]
Hydrothermal			
*o*-phenylenediamine and lysine	CDs-0-2.51 nm	Endoplasmic reticulum polarity	[160]
	CDs-1-2.55 nm		
	CDs-2-3.35 nm		
	CDs-3-2.95 nm		
	(NA)		
Hydrothermal			

NA: Not Available.

**Table 4:** CDs synthesized for dual-mode detections (PL and colorimetric).

Precursor and Synthesis Route	Size (QY)	Application	Ref
2,5-diaminobenzene sulphonic acid, 4-aminophenylboronic acid hydrochloride and Fe^3+^	NA (0.7%—in the absence of ascorbic acid, 2.3%—in the presence of ascorbic acid)	Detection of ascorbic acid	[165]
Hydrothermal			
Folic acid and p-phenylenediamine	2 nm (8.4%)	Detection of organophosphate pesticide	[166]
Hydrothermal			
Methylene-bis-acrylamide, *p*-phenylenediamine, and trifluoroacetic acid	3.9 ± 0.2 nm (7.5%)	Detection of Al^3+^	[167]
Hydrothermal			
m-phenylenediamine and citric acid	3–4 nm (65%)	Detection of Cr (VI)	[168]
Solvothermal			
Citric acid and ethylenediamine	NA	Detection of glucose	[169]
Microwave-assisted hydrothermal			
*N*-(phosphonomethyl)iminodiacetic acid (PMIDA) and branched PEI	6.71 nm (15.91%)	Detection of formaldehyde and bioimaging	[170]
Hydrothermal			

NA: Not Available.

**Table 5:** Carbon dot-based chemiluminescence detection.

Precursor and Synthesis Route	Size (QY)	Target of Detection	Ref
Glucose	4 nm (NA)	Gallic acid	[176]
Ultrasonic			
Ethylene glycol	5 ± 1 nm (NA)	Methoxyestradiol	[177]
Solvothermal			
L-cysteine and citric acid	3.1 nm (NA)	Carcinoembryonic antigen	[178]
Pyrolysis			
Citric acid and 1-3-(3,4-Dihyroxyphenyl) alanine (L-DOPA)	4.5 nm (NA)	Uric acid	[179]
Solid phase thermal			
Phloroglucinol	5.4 nm (NA)	Ascorbic acid	[180]
Solvothermal			
Citric acid, L-cysteine, and heteroatoms	10 nm (80%)	Oxytetracycline	[181]
Hydrothermal			

NA: Not available.

**Table 6:** Carbon dot-based electrochemiluminescence detection.

Precursor and Synthesis Route	Size (QY)	Application	Ref
Fullerene (C_60_)	3.5 ± 1 nm (NA)	Determination of microRNA-21	[188]
Hydrothermal			
Citric acid and L-cysteine	NA	Detection of atrazine	[189]
Pyrolysis			
Melamine	2 nm (NA)	Detection of butein	[190]
Hydrothermal			

NA: Not available.
